# Supplementary material for: Co-Stimulation of Purinergic P2X4 and Prostanoid EP3 Receptors Triggers Synergistic Degranulation in Murine Mast Cells
Source: Int J Mol Sci. 2019 Oct 17;20(20):5157. doi: 10.3390/ijms20205157 (PMC6829402; doi:10.3390/ijms20205157)
Supplement: Supplementary file 1 [file ijms-20-05157-s001.pdf]

Supplementary Fig. 1

The entire western blots shown in **Figure 5A**

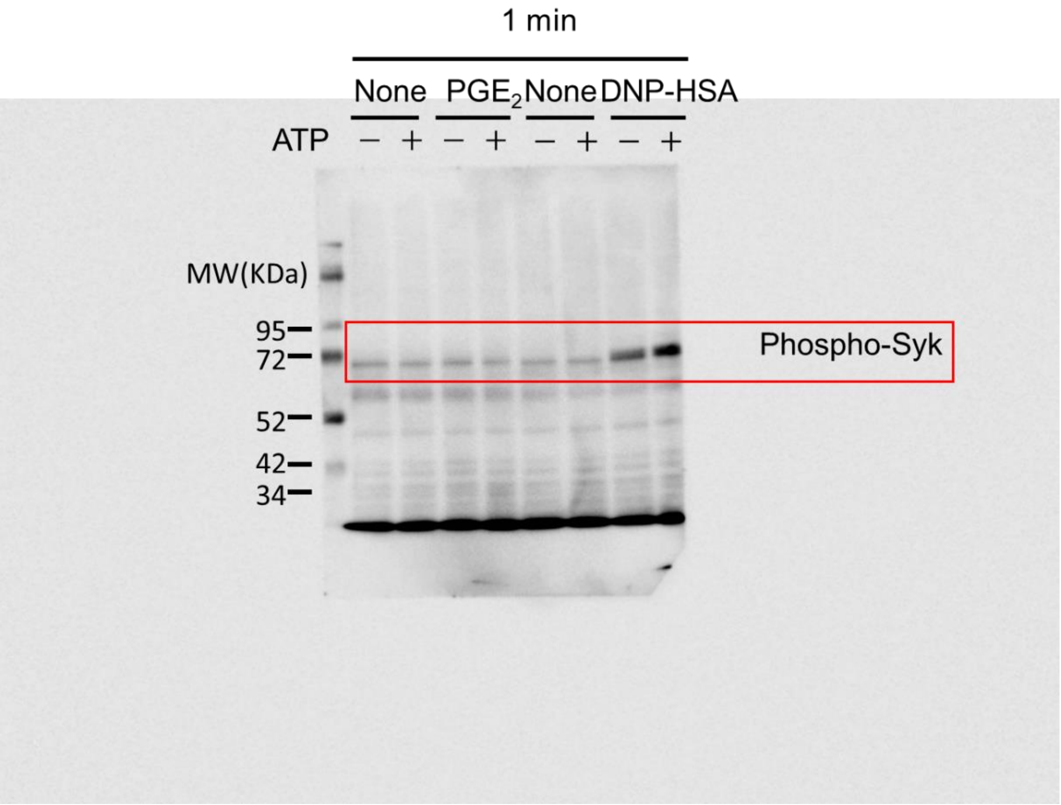

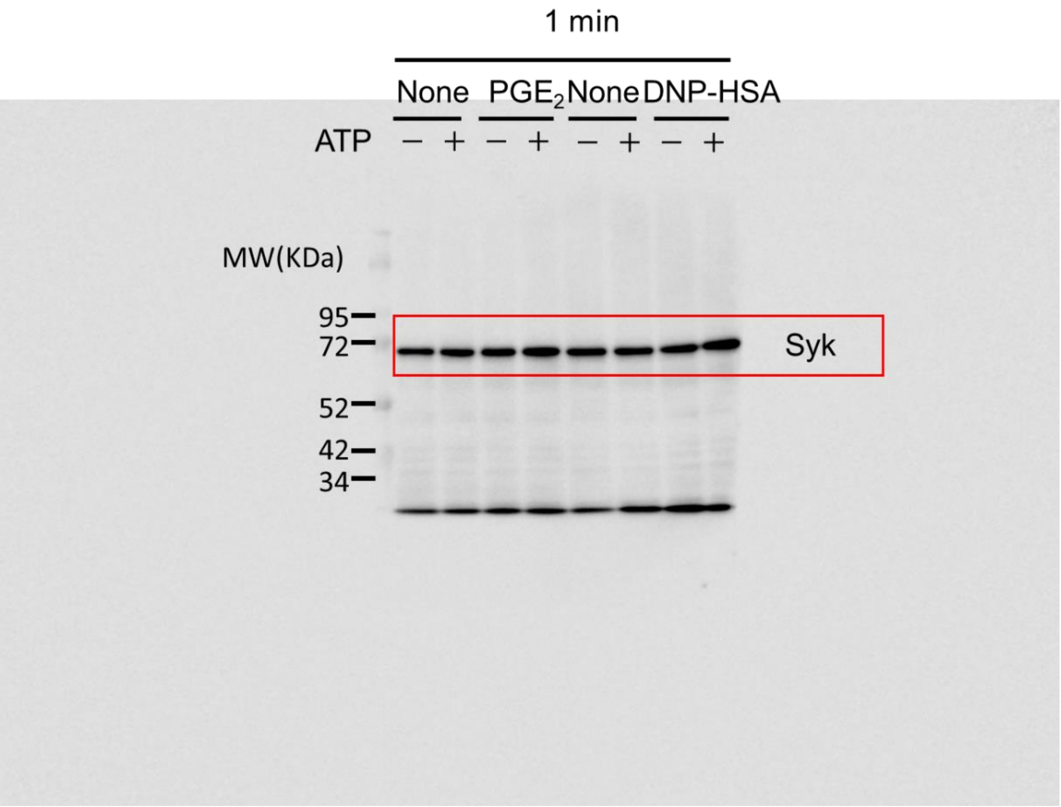

Supplementary Fig. 2

The entire western blots shown in **Figure 5B**

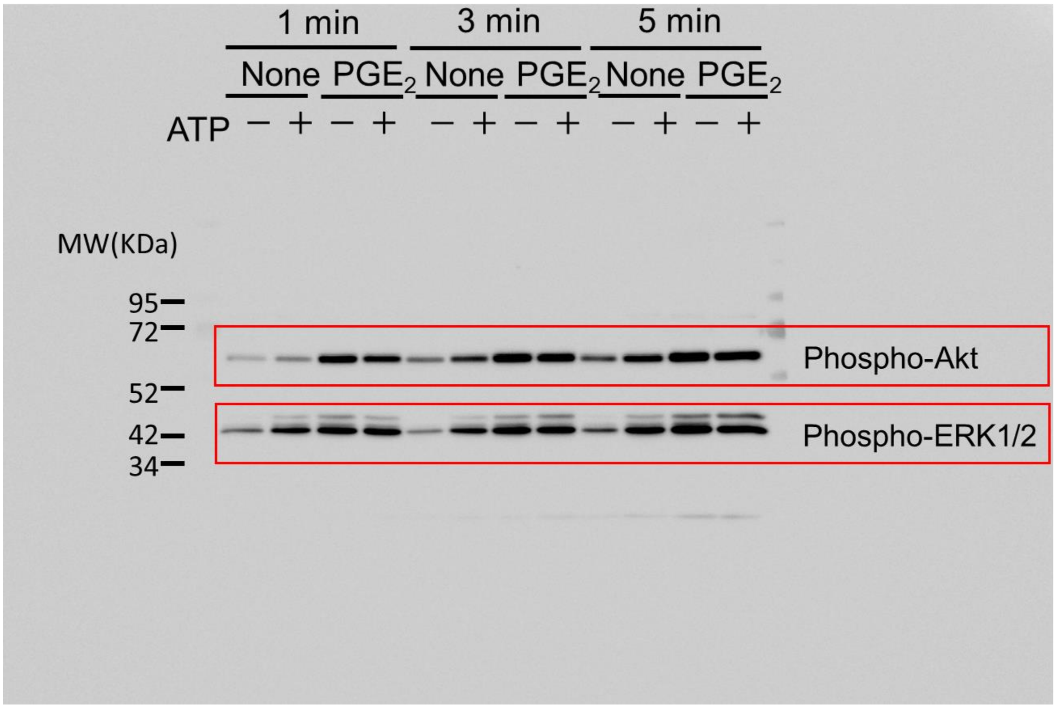

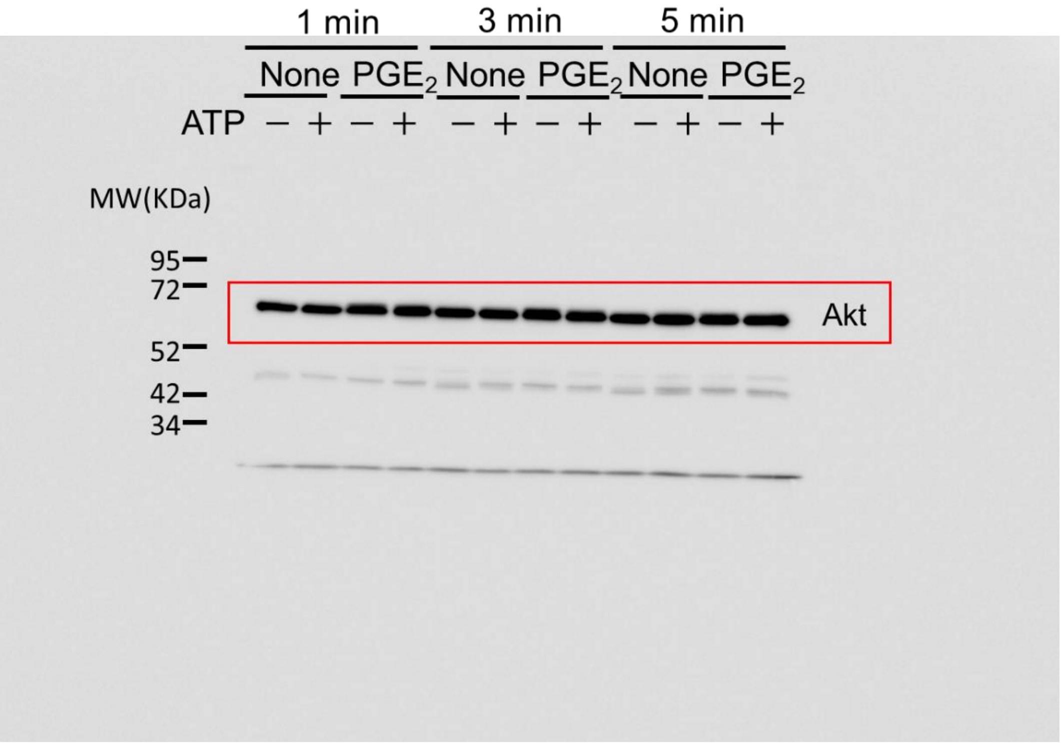

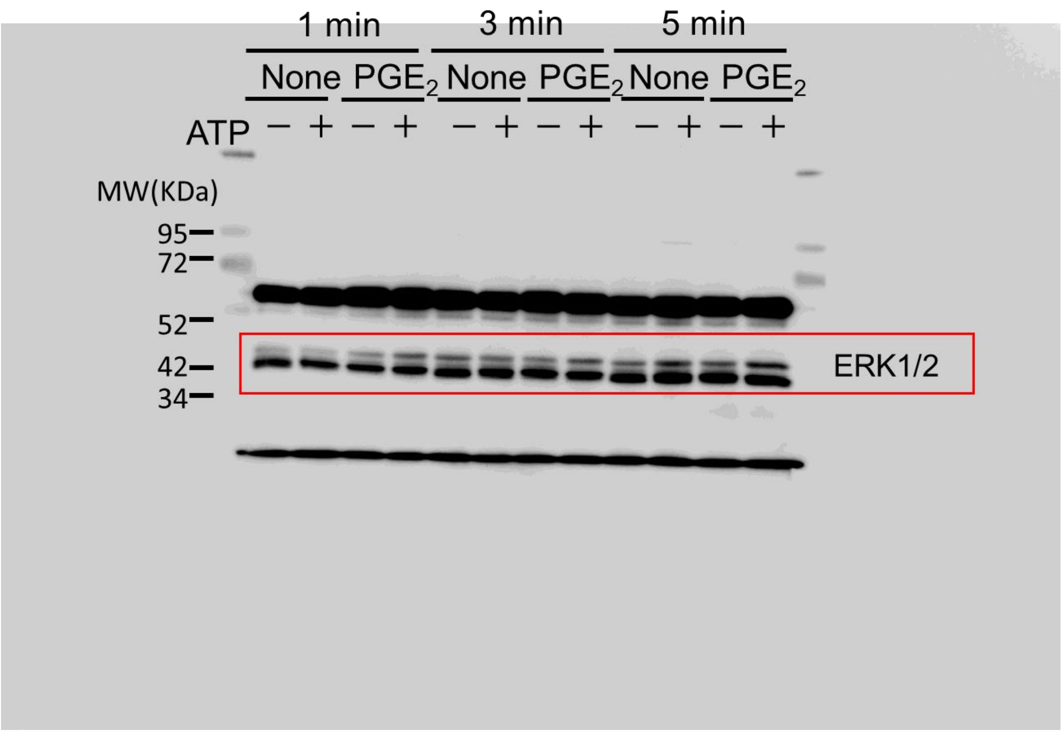
1 min
3 min
5 min
None
PGE2
None
PGE2
None
PGE2
ATP − + − + − + − + − + − +
MW(KDa)
95
72
52
42
34
ERK1/2
